# Influence of degree of specific allergic sensitivity on severity of rhinitis and asthma in Chinese allergic patients

**DOI:** 10.1186/1465-9921-12-95

**Published:** 2011-07-15

**Authors:** Jing Li, Ying Huang, Xiaoping Lin, Deyu Zhao, Guolin Tan, Jinzhun Wu, Changqing Zhao, Jing Zhao, Michael D Spangfort, Nanshan Zhong

**Affiliations:** 1 State Key Laboratory of Respiratory Disease, The First Affiliated Hospital, Guangzhou Medical College, Guangzhou, Guangdong, China; 2 The Children's Hospital, Chongqing University of Medical Sciences, Chongqing, China; 3 The General Hospital of Shenyang Military Command, Shenyang, Liaoning, China; 4 Nanjing Children's Hospital, Nanjing, Jiangsu, China; 5 The Third Hospital of Xiangya Medical University, Changsha, Hunan, China; 6 The First Hospital of Xiamen, Xiamen, Fujian, China; 7 The Second Hospital of Shanxi Medical University, Taiyuan, Shanxi, China; 8 Capital Institute of Pediatrics, Beijing, China; 9 ALK-Abello A/S, Asia Pacific Region, Hongkong, China

**Keywords:** sensitization, aeroallergens, disease severity, allergic rhinitis, asthma, association.

## Abstract

**Background:**

The association between sensitizations and severity of allergic diseases is controversial.

**Objective:**

This study was to investigate the association between severity of asthma and rhinitis and degree of specific allergic sensitization in allergic patients in China.

**Method:**

A cross-sectional survey was performed in 6304 patients with asthma and/or rhinitis from 4 regions of China. Patients completed a standardized questionnaire documenting their respiratory and allergic symptoms, their impact on sleep, daily activities, school and work. They also underwent skin prick tests with 13 common aeroallergens. Among the recruited subjects, 2268 provided blood samples for serum measurement of specific IgE (sIgE) against 16 common aeroallergens.

**Results:**

Significantly higher percentage of patients with moderate-severe intermittent rhinitis were sensitized to outdoor allergens while percentage of patients sensitized to indoor allergens was increased with increasing severity of asthma. Moderate-severe intermittent rhinitis was associated with the skin wheal size and the level of sIgE to *Artemisia vulgaris *and *Ambrosia artemisifolia *(p < 0.001). Moderate-severe asthma was associated with increasing wheal size and sIgE response to *Dermatophagoides *(*D*.) *pteronyssinus *and *D. farinae *(p < 0.001). Moderate-severe rhinitis and asthma were also associated with increase in number of positive skin prick test and sIgE.

**Conclusions:**

*Artemisia vulgaris *and *Ambrosia artemisifolia *sensitizations are associated with the severity of intermittent rhinitis and *D. pteronyssinus *and *D. farinae *sensitizations are associated with increasing severity of asthma in China. Increase in number of allergens the patients are sensitized to may also increase the severity of rhinitis and asthma.

## Background

The prevalence of asthma and allergic rhinitis symptoms varies considerably across the world [1,2]. In China, the prevalence of allergic rhinoconjunctivitis symptoms varies from 8.7 to 24.1% documented by self-reported telephone interviews conducted between 2004 and 2005 in 11 cities [3]. The prevalence of respiratory allergy is increasing in China [3,4] and an international comparative study found that in the city of Guangzhou, the prevalence of asthma symptoms among children aged 13-14 years increased from 3.4% in 1995 to 4.8% in 2001 [4] and to 6.1% in 2009 (unpublished data).

Atopic sensitization is a risk factor for the development of upper and lower respiratory symptoms [5,6]. Exposure to allergens the patients are sensitized to may exacerbate symptoms of rhinitis and asthma by promoting airway inflammation, airflow limitation, and airway hyperreponsiveness (AHR). Sensitization to indoor allergens correlates well with indoor allergen exposure in pre-school and school-age children [7,8]. Furthermore, exposure and sensitivity follows a dose-dependent relationship [9]. Evidence supporting this relationship is particularly strong for house dust mite (HDM) sensitization [9]. Allergic rhinitis can also be caused by pollens from grasses and trees which are the most important sources of outdoor sensitizing allergens [10,11]. We have previously performed an epidemiological study of the prevalence of sensitization in patients with asthma and/or rhinitis in mainland China [12]. For indoor and outdoor allergens, we found that house dust mite sensitization was consistently associated with asthma whereas *Artemisia vulgaris *and *Ambrosia artemisifolia *pollen sensitizaions were associated with the development of rhinitis [12].

Both rhinitis and asthma are diseases of variable severity. Many studies have shown that the degree of allergic sensitivity as reflected by elevated serum allergen-specific IgE levels or allergen skin wheal size is related to asthma severity [13,14], however, other studies [15,16] did not find this relationship.

Thus, the influence of the degree of allergic sensitivity on the disease severity of allergic asthma and rhinitis remains uncertain. The aim of this study was to investigate the relationship between size of skin test or level of serum specific IgE and the severity of asthma and rhinitis in Chinese patients based on data from a recently conducted nation-wide multicentre epidemiology study.

## Methods

### Study population and definitions

The study was a cross-sectional epidemiologic survey, conducted from February 2006 to March 2007 in 17 cities with 24 participating centers from northern, eastern, south western and southern coastal regions of China. The study covered mid-temperate, warm-temperate, subtropical and tropical zones of China. Patients aged 5 to 65 years attending outpatient clinics at 24 centers, and diagnosed as rhinitis and/or asthma, were invited to participate in this survey. By evaluating their history, questionnaire and relevant tests, rhinitis was defined as having symptoms of sneezing, or a running, itchy or blocked nose when the patient did not have a cold or flu. Asthma was defined by a history of recurrent dyspnea, wheezing or cough episodes, positive airway reversibility testing (FEV_1 _increasing ≥12% and 200 ml after inhalation of 400 mg of salbutamol) or positive airway responsiveness testing (FEV_1 _decreasing ≥20% when ≤ 7.8 μmol of cumulative dose of histamine is administered). The study was approved by the Ethics Review Board of each study center and all patients gave written consent before the study.

### Questionnaire

The standardized questionnaire was administered by the trained physicians or research nurses face-to-face with questions regarding demographic characteristics, family history of allergic diseases, symptoms of rhinitis, wheezing or coughing, eczema and burning or itchy eyes, smoking habits, environmental exposure factors, animal pet ownership and dietary habits. Questions about impact of allergic symptoms on daily activities, work or school, night-time sleep, and use of medications for controlling the symptoms were also documented.

### Assessment of severity of rhinitis and asthma

According to the Allergic Rhinitis and its Impact on Asthma guidelines [17], rhinitis was classified as "mild" and "moderate/severe" depending on the severity of symptoms and their impact on sleep, daily activities, school and work evaluated by the questionnaire. Severity of asthma was classified according to the 2006 version of Global Initiative for Asthma guidelines [18].

### Skin prick test (SPT)

The sensitivity to thirteen common aeroallergens was tested including *Dermatophagoides (D.) pteronyssinus, D. farinae and Blomia tropicalis*, dog, cat, *Periplaneta americana*, *Blatella germanica, Artemisia vulgaris, Ambrosia artemisifolia*, mixed grass and tree pollen, mould mix I and IV. Allergen extracts and control solutions were obtained from ALK (Horsholm, Denmark). Histamine (10 mg/ml) and diluent were used as positive and negative controls. SPT was performed on the volar side of the forearm. The wheal reaction after 15 minutes was measured as the mean of the longest diameter and the length of the perpendicular line through its middle. A positive skin reaction was defined as a wheal size 3 mm greater than the negative control. The result was also expressed as skin index (SI = mean size of allergen wheal/mean size of histamine wheal). Atopy was defined as the presence of at least one positive skin reaction to any allergen tested.

We originally recruited 6411 questionnaires and 6393 skin test reports. Among the 6411 questionnaires, 107 were invalid for lacking proper diagnosis, incompletely answering the questionnaire or missing skin test report. Of the 6393 skin test reports, 89 were rejected for missing questionnaire data, wrong codings, or missing the histamine and normal saline readings. Hence, we restricted our final valid data with 6304 patients.

### Serum specific IgE Analysis

Among the 24 centers, 14 of them obtained serum samples from their subjects for sIgE analysis. With the written consents, peripheral blood was obtained from patients in the above centers only after completing the questionnaires and skin prick tests. Finally, 2268 out of the 6304 patients (806 with rhinitis alone, 773 with asthma alone and 689 with both rhinitis and asthma) from four regions provided blood for measurement of serum allergen-specific IgE (sIgE). Ten ml of blood from each subject was coagulated at room temperature, centrifuged, stored at -20°C. The sIgE against *D. pteronyssinus, D. farinae*, cat, dog, *Periplaneta americana*, *Blatella germanica, Penicillium, Cladosporium, Fusarium*, sycamore, willow, cottonwood, elm, grass pollen, *Artemisia vulgaris, Ambrosia artemisifolia *was measured with the ADVIA Centaur^® ^immunoassay system (Bayer Healthcare LLC, Tarrytown New York, USA) [19]. The analysis for sIgE was defined to be positive if the measurement was ≥ 0.35 kU/L.

### Quality control

Standardized protocol, questionnaire, allergen skin prick testing set, and operating procedures were used by all the centers. All questionnaire interviewers and performers of skin prick testing were trained before the study. Results of questionnaire and skin prick tests were sent every month to Guangzhou, where the data were input and analyzed. Quality control reports were then prepared for each center. Each completed questionnaire and skin test report was verified by the center supervisor and the results were double-checked by the principal investigator and fed back to each center. All questionnaires and skin test data were coded and input into a programmed database by two persons independently. The entered data were checked for out-of-range values and logic mistakes.

### Statistical analysis

For all analyses p < 0.05 was regarded as statistically significant. Prevalences of sensitization to various groups of allergens are presented. The differences of the sensitization rate between different severities of rhinitis and asthma were determined by chi-square tests. Skin prick test mean wheal diameter were used as raw data. The relationship between quantitative mean skin wheal diameter and severity of rhinitis or asthma was analyzed using logistic regression. Fitted predicted probability curves of moderate-severe rhinitis and asthma according to the wheal size of skin sensitizations were plotted using the results from the logistic regression. For the quantitative evaluations, the OR are presented for different skin prick test mean wheal diameters expressing the increased risk of severity of rhinitis and asthma associated with increasing skin wheal size. For associations between sIgE concentrations and different severities of rhinitis and asthma, we calculated the prevalence of rhinitis and asthma severities with different sIgE levels against *D. pteronyssinus, D. farinae, Artemisia vulgaris *and *Ambrosia artemisifolia *sIgE and the statistical significance of the differences were determined by using chi-square tests. All data were categorized and analyzed using the Statistical Package for the Social Sciences (SPSS Inc. Chicago, IL, USA) for Windows Release 13.0 and Microcal Origin 6.0 (Microcal Software Inc., Northampton, MA, USA).

## Results

Of the 6304 patients, 967 subjects had mild intermittent rhinitis, 452 had moderate-severe intermittent rhinitis, 1729 had mild persistent rhinitis and 1154 had moderate-severe persistent rhinitis. Asthma was under control in 741 patients while 441 patients had intermittent asthma (step 1), 735 with mild persistent (step 2), 948 with moderate persistent (step 3) and 915 with severe persistent asthma (step 4). Patients with moderate-severe intermittent rhinitis had significantly higher prevalence of sensitization to dogs, *Artemisia vulgaris, Ambrosia artemisifolia*, mixed grass pollen and mixed tree pollen (p < 0.001) by skin prick tests. They also showed significantly greater percentage of multiple sensitizations (p < 0.05). Prevalence of sensitization to *D. pteronyssinus, D. farinae, blomia tropicalis*, dog and cat was increased with increasing of disease severity in patients with asthma. Furthermore, with increasing severity of asthma, there is higher proportion of patients with multiple sensitizations (Table 1).

**Table 1:** Prevalence (%) of allergen skin sensitizations in patients with rhinitis and asthma of different severity

	Rhinitis						Asthma					
	
	MI (n = 967)	MSI (n = 452)	MP (n = 1729)	MSP (n = 1154)	χ2	p	MI (n = 441)	MIP (n = 735)	MOP (n = 948)	SP (n = 915)	χ2	p
D. pteronyssinus	61.1	59.7	61.8	60.9	7.0	0.092	51.7	58.5	62.0	64.5	34.6	0.000
D. farinae	62.1	60.6	64.3	63.1	3.16	0.371	52.6	60.2	63.7	65.6	36.5	0.000
Blomia tropicalis	43.3	43.3	44.4	44.9	0.6	0.901	32.3	39.0	44.4	48.4	56.7	0.000
Dog	15.9	20.1	14.0	16.5	13.8	0.003	12.4	13.2	14.3	18.0	17.2	0.001
Cat	9.8	13.1	10.5	10.1	5.03	0.17	8.7	11.7	12.9	13.6	11.0	0.012
American cockroach	15.3	16.7	16.9	17.2	0.88	0.83	15.8	17.4	16.6	19.4	5.8	0.12
Blatella germanica	10.7	13.7	12.8	13.0	2.53	0.47	9.9	10.6	13.3	13.2	8.2	0.042
Artemisia vulgaris	16.5	30.5	8.0	13.1	202.4	0.000	9.8	7.7	9.6	8.8	2.3	0.52
Ambrosia artemisifolia	10.9	20.1	3.9	6.9	167.7	0.000	5.2	4.7	5.2	4.1	2.5	0.483
^1^Mixed grass pollen	3.1	4.8	2.0	1.5	21.0	0.000	2.4	2.1	2.7	2.3	0.7	0.872
^2^Mixed tree pollen	4.6	8.2	2.2	3.8	45.5	0.000	4.9	2.1	3.2	3.9	7.7	0.053
^3^Mixed mould I	6.9	6.3	7.8	6.2	7.6	0.07	6.2	7.7	8.1	8.2	2.9	0.409
^4^Mixed mould IV	5.4	5.0	5.9	4.1	6.0	0.13	4.8	4.2	6.0	5.5	2.9	0.404
≥2 sensitizations	72.6	74.3	68.5	69.5	9.548	0.023	59.4	65.3	70.3	71.1	34.24	0.000

^1 ^Mixed grass pollen: Dactylis glomerata, Festuca pratensis, Lolium perenne, Phleum pretense and Poa pratensis; ^2 ^Mixed tree pollen: Ulmus americana, Platanus acerifolia, Salix caprea and Populus deltoids; ^3 ^Mixed mould I: Alternaria alternata, Cladosporium herbarum, Fusarium sp and Chaetomium globosum; ^4 ^Mixed mould IV: Penicillium (P.) brevicompactum, P. expansum, P. notatum and P. roqueforthi. MI: mild intermittent; MSI: moderate-severe intermittent; MP: mild persistent; MSP: moderate-severe persistent; MIP: mild persistent; MOP: moderate persistent; SP: severe persistent.

Serum specific IgE against 16 common aeroallergens was measured in 2268 patients in whom 175 were classified as mild intermittent rhinitis, 281 as moderate-severe intermittent rhinitis, 596 as mild persistent rhinitis and 339 as moderate-severe persistent rhinitis. For asthma patients, 405 were at mild intermittent stage, 313 at mild persistent, 335 at moderate persistent and 628 at severe persistent stage. *D. pteronyssinus and D. farinae *were found to be the most prevalent allergens followed by *Artemisia vulgaris *and *Ambrosia artemisifolia *with sIgE measurements in patients with rhinitis and asthma. Significantly higher percentage of patients with moderate-severe intermittent rhinitis was sensitized to *Artemisia vulgaris *(p < 0.001), *Ambrosia artemisifolia *(p < 0.001), willow (p < 0.01), elm (p < 0.05) and grass pollen (p < 0.05). Elevated levels of sIgE against *D. pteronyssinus *and *D. farinae *in patients with asthma were associated with increasing the severity (p < 0.001). Multiple sensitizations was significantly associated with increasing in level of asthma severity (p < 0.001) (Table 2).

**Table 2:** Prevalence (%) of serum specific IgE positivity to tested allergens in patients with rhinitis and asthma of different severity

	Rhinitis						Asthma					
	
	MII (n = 175)	MSI (n = 281)	MIP (n = 596)	MSP (n = 339)	χ2	p	MII (n = 405)	MIP (n = 313)	MOP (n = 335)	SEP (n = 628)	χ2	p
D. pteronyssinus	44.3	45.2	50.2	47.0	2.96	0.401	22.7	52.4	59.4	79.1	332.6	0.000
D. farinae	43.8	46.0	49.1	44.7	2.56	0.463	21.0	49.2	58.5	77.2	320.2	0.000
Cat	10.2	7.8	9.1	10.1	1.17	0.765	7.3	9.8	9.5	10.5	1.90	0.594
Dog	5.4	3.9	4.2	5.2	1.03	0.792	2.1	4.4	3.0	5.1	4.86	0.182
American cockroach	0	1.6	1.3	0.6	3.13	0.372	2.3	1.1	1.1	1.8	1.46	0.712
Blatella germanica	0	3.5	2.2	2.3	5.75	0.125	1.6	2.9	4.0	2.3	3.68	0.302
Penicillium	2.1	0.9	1.3	0.3	3.40	0.337	0	0.6	1.2	1.3	4.38	0.209
Cladosporium	0	0	0.2	0	1.26	0.741	0	0	0	0.3	1.62	0.795
Fusarium	29.1	28.2	23.9	24.2	2.29	0.515	17.9	22.6	25.7	22.7	2.534	0.470
Sycamore	5.1	5.6	2.1	3.3	7.57	0.060	2.3	2.2	1.8	2.7	0.741	0.867
Willow	3.8	6.8	2.0	4.0	11.9	0.008	1.7	1.6	2.9	2.9	1.773	0.642
Cottonwood	5.0	6.8	2.7	4.5	7.83	0.050	2.8	2.1	3.6	3.4	0.998	0.805
Elm	6.8	9.1	4.1	4.5	9.63	0.024	2.2	2.1	3.1	4.0	2.837	0.416
Grass pollen	4.3	4.4	1.3	2.8	8.87	0.030	1.1	0	1.9	2.1	4.55	0.212
Artemisia vulgaris	19.5	27.1	6.1	6.7	93.2	0.000	5.4	4.0	4.8	5.4	.747	0.870
Ambrosia artemisifolia	14.9	29.2	0.7	0.9	98.7	0.000	0	0	0.7	1.2	4.651	0.184
≥2 sensitizations	16.3	21.8	19.9	21.8	7.30	0.063	15.8	18.8	21.7	26.6	39.1	0.000

MII: Mild intermittent; MSI: Moderate-severe intermittent; MIP: Mild persistent; MSP: Moderate-severe persistent; MOP: Moderate persistent; SEP: Severe persistent

### Allergen skin test sizes and severity of rhinitis and asthma

**Figure 1:**
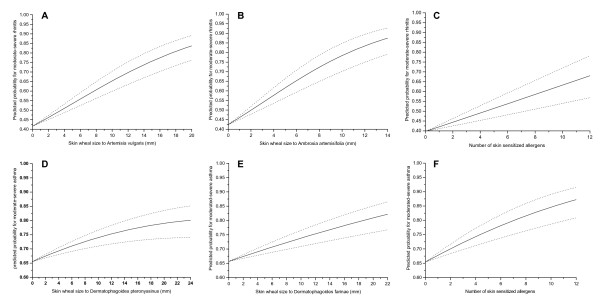
**Skin allergen sensitization and severity of rhinitis and asthma**. (A-F) Fitted predicted probability curves (and 95% CI) for moderate-severe rhinitis at given skin wheal size to Artemisia vulgaris (A), Ambrosia artemisifolia (B) and number of skin sensitized allergens (C), derived from the logistic regression analysis. Fitted predicted probability curves (and 95% CI) for moderate-severe asthma at given skin wheal size to Dermatophagoides pteronyssinus (D), Dermatophagoides farinae (E) and number of skin sensitized allergens (F), derived from the logistic regression analysis.

Using allergen skin prick test wheal size as a continuous variable, the risk of having moderate-severe rhinitis in our patients was at around 40%-42.5% when they were not sensitized to *Artemisia vulgaris *(Figure 1A) or *Ambrosia artemisifolia *(Figure 1B), or any tested allergen (Figure 1C). But the risk increased significantly with increasing skin wheal size to *Artemisia vulgaris *(OR 1.12, 95% CI 1.07-1.14, p < 0.001) and *Ambrosia artemisifolia *(OR 1.19, 95% CI 1.13-1.41, p < 0.001) corresponding to OR of 4.29 and 4.85 at 10 mm wheal size, and 11.52 and 23.71 at 20 mm, respectively (Figure 1A-1B). Similarly, when patients were not sensitized to *D. pteronyssinus *(Figure 1D) and *D. farinae *(Figure 1E), or to the tested allergens (Figure 1F), the probability of having moderate-severe asthma was at around 66%, but the risk increased for 1.21-fold per mm increase in skin wheal size to *D. pteronyssinus *and *D. farinae *(95% CI 1.09-1.46 and 1.10-1.47 respectively, p < 0.001), corresponding to an OR of 1.84 and 1.74 at 10 mm wheal size, and 2.76 and 2.63 at 20 mm, respectively (Figure 1D-1E). In addition, moderate-severe rhinitis and asthma were also associated with increasing number of skin sensitized allergens (Figure 1C, F).

### Allergen sIgE levels and severity of asthma and rhinitis

**Figure 2:**
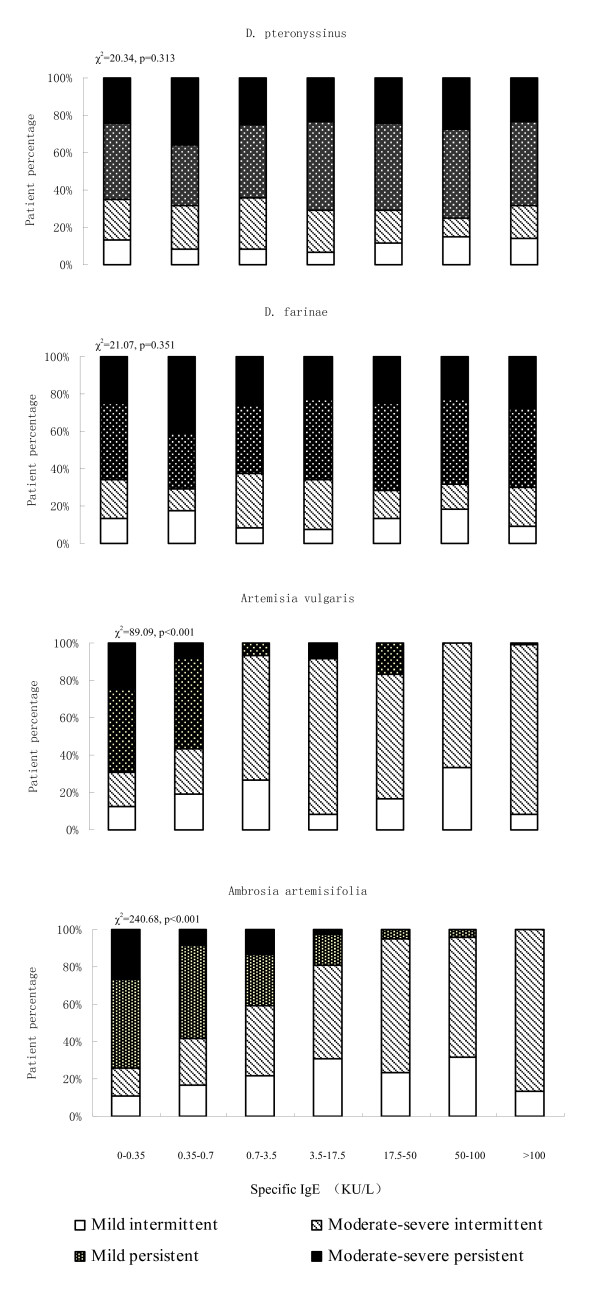
**Serum specific IgE levels and severity of rhinitis**. Distributions in percentage of patients with mild intermittent, moderate-severe intermittent, mild persistent and moderate-severe persistent rhinitis by different levels of serum specific IgE.

**Figure 3:**
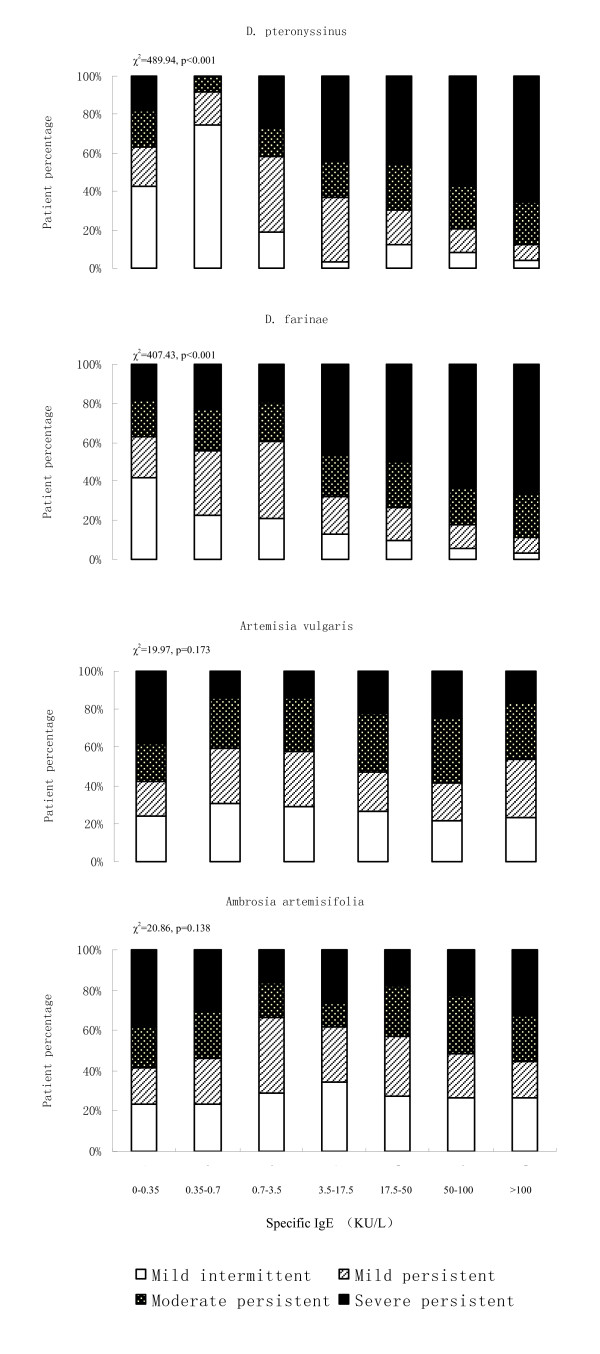
**Serum specific IgE levels and severity of asthma**. Distributions in percentage of patients with mild intermittent, mild persistent, moderate persistent and severe persistent asthma by different levels of serum specific IgE.

Among patients with rhinitis, we found that significantly higher percentage of patients with moderate-severe intermittent rhinitis had higher level of sIgE to *Artemisia vulgaris *and *Ambrosia artemisifolia *(p < 0.001) but not to *D. pteronyssinus *and *D. farinae *(Figure 2). For asthma patients, sIgE levels against *D. pteronyssinus *and *D. farinae*, but not *Artemisia vulgaris *and *Ambrosia artemisifolia*, were significantly associated with increasing of asthma severity (p < 0.001) (Figure 3).

## Discussion

In this nation-wide multicentre epidemiologic study of more than 6300 asthmatic and rhinitis patients with varying disease severity in China, we found *D. pteronyssinus *and *D. farinae *sensitizations were significantly associated with severity of asthma while *Artemisia vulgaris *and *Ambrosia artemisifolia *sensitizations were related to severity of rhinitis. Furthermore, multiple allergen sensitization was also associated with severity of rhinitis and asthma as determined by either skin prick test or sIgE measurements.

In this paper, our data show that severity of asthma was significantly correlated with skin index of reactivity to *D. pteronyssinus, D. farinae and Blomia tropicalis*. Furthermore, we also found that elevated levels of sIgE to *D. pteronyssinus *and *D. farinae *correlate significantly with increasing severity of asthma. Our findings support the concept that sensitization against indoor allergens may affect asthma severity [13,20]. Allergens induce sensitizations in persons who are in high risk and repetitive exposure to the allergens may lead to allergic inflammatory reactions in the airway mucosa [21]. Airway inflammation may be variably associated with changes in airway hyperresponsiveness, airflow limitation, respiratory symptoms, and disease chronicity [22]. Our finding that patients who had HDM sensitization were more likely to have more severe asthma, compared to those without sensitization, is consistent with many other studies in children or adults [23]. Platts-Mills et al. [24] reported that load of house dust mites is associated with the onset of respiratory allergic conditions, especially bronchial asthma, and that there exists a threshold of HDM exposure to induce symptoms of asthma. Even exposure to low levels of mite allergens (0.02-2.0 μg/g dust) was found to be a significant risk factor for sensitization [25]. A few studies found several species of HDM in indoor environment in China [26,27] and relatively high levels of HDM group 1 allergens (> 10 μg/g dust) has been detected in a very high proportion of dust samples from southern China [28].

Not surprisingly, we demonstrated quantitative association between the size of skin test and the specific IgE levels to pollens especially *Artemisia vulgaris *and *Ambrosia artemisifolia *and moderate-severe intermittent rhinitis. Although we did not analyze the data by stratification of the patients with regions and seasons in this paper, we predict that these patients are mainly from the northern parts of China undergoing clinical sampling during the season from July to September [12]. One recently published study [11] demonstrated that sIgE levels to birch- and grass-pollen at baseline as well as during the pollen season were associated with seasonal symptom severity of rhinitis and use of rescue medications. In contrast, adult patients with seasonal allergic rhinitis have been investigated by several studies in this respect. Some investigators found a positive association between sIgE levels and clinical symptoms [29,30], although symptoms were also dependent on other factors, such as the ease of histamine release by basophils. Other studies did not find strong associations or reported inconsistent findings [31,32]. This inconsistency may be explained by differences in allergens, age or other characteristics of the patient populations studied. At least this seems to be the reason for a marked variability in the outcome of a variety of studies investigating the capacity to predict symptomatic allergy from sIgE levels in children [33]. We therefore assume that some of the above-mentioned differences among studies in respiratory allergies may be explained by the varying parameters of the allergens studied, the age of the patients and the measurements of clinical disease severity.

Surprisingly, we failed to find the relationship between HDM skin test size and specific IgE levels and severity of any type of rhinitis, especially persistent rhinitis, however, our finding supports the facts that outdoor allergens affect rhinitis significantly [13,20]. Many studies have shown that pollen such as *Artemisia vulgaris *and *Ambrosia artemisifolia *is a larger allergen compared with HDMs and is mainly deposited in the upper airway where it induces local inflammatory or pathological changes, whereas enzymatic activity of pyroglyphid mites seems to be important in the pathogenicity of lower airway and systemic inflammations [34,35]. We have extended this observation by demonstrating the same associations for Chinese weed grass pollens *Artemisia vulgaris *and *Ambrosia artemisifolia *within the group of patients defined as atopic using standard definitions [17]. These findings also indicate that IgE-mediated sensitization is not dichotomous in its relation to the expression, severity and temporal pattern of upper and lower respiratory allergic diseases.

In this study, we also found by both skin test and sIgE measurements that patients with sensitizations to multiple allergens were significantly more likely to have more severe rhinitis and asthma. Our results are in agreement with the study by Simpson et al. [36]. They investigated a group of adults with asthma showing that sensitization to dust mite, cat, dog, and mixed grasses as well as multiple sensitizations were all independently associated with asthma. The data of another study [13] suggested that the development of specific IgE response to multiple indoor allergens is an important factor in the persistence of bronchial obstruction in children with asthma.

In summary, the results of the current study emphasize the importance of sensitization to indoor allergens in asthma severity and to outdoor allergens in severity of rhinitis. Sensitization to more than one allergenic source also significantly increases the possibility of developing moderate-severe rhinitis and asthma.

## Competing interests

The authors declare that they have no competing interests.

## Authors' contributions

JL mainly designed the study, performed the survey, collected the data, performed the statistical analysis and the drafted the manuscript. YH participated in designing the study, performed the survey, collected the data and drafted the manuscript. XL participated in designing the study, performed the survey, and collected the data. DZ participated in designing the study, performed the survey and collected the data. GT performed the survey, collected the data. JW participated in designing the study, performed the survey and collected the data, HZ performed the survey and collected the data. JZ participated in designing the study, performed the survey and collected the data. MS designed the study, performed the statistical analysis and the drafted the manuscript. NZ mainly designed the study, performed the statistical analysis and the drafted the manuscript. All members of China Alliance of Research on Respiratory Allergic Disease participated in discussion the protocol of the study, perform the survey and collected the data. All authors read and approved the final manuscript.
